# Molecular Mechanisms of Neural Plasticity: From Basic Research to Implications for Visual Functional Rescue

**DOI:** 10.3390/ijms232113183

**Published:** 2022-10-29

**Authors:** Alessandro Sale

**Affiliations:** Neuroscience Institute of the National Research Council of Italy (CNR), Via Moruzzi 1, 56124 Pisa, Italy; alessandro.sale@in.cnr.it

Visual system plasticity, the capability of visual connections to modify their structure and function in response to experience, is an essential property underlying the maturation of visual functions during development, behavioral flexibility in response to subtle environmental changes, and adaptive repair in conditions of disease or trauma [1,2,3].

Given the centrality of the visual system as a paradigmatic model of brain plasticity and considering the relevance of vision and visual disorders for human life, it is not surprising that the neural mechanisms of plasticity within the visual system have been a long-term target of research, starting from the pioneering work of Hubel and Wiesel. The intention of this Special Issue is to highlight the results of novel research focused on the mechanisms underlying visual system plasticity, both during the critical period and in the adult brain, with a special consideration for potential translational applications of the results achieved by basic research studies. 

Zloh et al. [4] concentrated on an intriguing phenomenon described in the retina of mammals. Müller cells, a dominant form of neuron-supporting macroglial cells in the retina [5], have been previously reported to elicit retinal regeneration in response to daily visual stimulation in the critical period, acting through BDNF-mediated pathways in the mouse retina. The authors expanded this observation, performing a thorough analysis both in vivo and in vitro in the effort to provide a better characterization of the mechanisms underlying the reaction of Müller cells to visual stimulation. They examined whether high-contrast stimulation for two weeks was able to affect the expression of the neurotrophic factor BDNF and its inducible factor, VGF, and then investigated possible alterations at the level of pro-inflammatory mediators. The results demonstrate that high-contrast stimulation activates the neurotrophic and neuroprotective properties of Müller cells, suggesting a possible direct role for this class of cells in retinal neuronal survival and improved functional outcomes in response to visual stimulation.

Pathologies affecting the integrity of retinal tissues are particularly relevant for their impact on vision. Stofkova et al. [6] focused on a form of autoimmune uveitis, an ocular inflammatory disease believed to derive from an abnormal activation of ocular antigen-specific CD4+ T helper cells in the eye. Using Gnat1rd17 mice, a model of late-onset rod-cone dystrophy displaying night blindness and experimental autoimmune uveoretinitis, the authors used a combination of transcriptome profiling, target identification, and functional validation to show that the prime alterations affecting the retina of Gnat1rd17 mice are mostly present at the level of their retinal dopaminergic system. Remarkably, these alterations were counteracted by L-DOPA administration, performed either systemically or locally, and able to suppress a pro-inflammatory phenotype in retinal endothelial cells by inhibiting NF-κΒ and STAT3 activity. Still in the field of retinal degeneration, Pietra et al. [7] performed a novel study on an experimental model of retinitis pigmentosa, a group of inherited disorders affecting 1 in 4000–5000 people worldwide and characterized by the progressive breakdown and loss of retinal photoreceptors, which eventually causes a loss of night vision, narrowing of the visual field, dyschromatopsia, and a decline in visual acuity [8]. While, so far, most studies have focused on retinal, thalamic, and collicular dysfunctions in both animal models and patients with retinitis pigmentosa, research aimed at analyzing the functional arrangement of the visual cortex in these conditions remains scarce [9]. The authors filled this gap, investigating visual cortical processing in rd10 mice following the progression of retinal deterioration. Strikingly, a strong alteration of the intracortical excitation/inhibition balance was evident in the visual cortex alongside photoreceptor degeneration. Perhaps surprisingly, antagonizing GABAergic neurotransmission with the non-competitive GABAA antagonist pentylenetetrazole further deteriorated visual functions, leading to the conclusion that the increased inhibitory drive in the visual cortex of rd10 mice might serve as a compensatory mechanism to increase the signal-to-noise ratio of the degraded sensory input originating from the degenerating retina. These novel observations provide a useful hypothesis to be tested in patients with retinitis pigmentosa, underscoring the need for future careful analyses focused on visual cortical changes as well.

The results of the studies by Zloh et al., Stofkova et al., and Pietra et al. emphasize the importance of the retina as a key model to investigate neural mechanisms underlying visual physiology and pathology. The retina has long been considered much less plastic than the cortex or the hippocampus, the very sites of experience-dependent neural plasticity. This traditional view, however, has been challenged by increasing evidence showing that retinal development can be modulated by either reductions or increments in the sensory inputs, as in the case of dark rearing or exposure to early environmental enrichment conditions. Strettoi et al. [10] provided an authoritative review article on the emerging role of retinal plasticity. In this work, the hippocampal and cortical paradigms of plasticity were compared with retinal structural and functional rearrangements in response to conditions of altered development or the occurrence of genetic diseases leading to neuronal degeneration. As emphasized by the authors, their review lends support to the fascinating theory by which a balanced level of moderated plasticity might be essential in an organ that is highly specialized for a dominant sensory modality that requires elevated levels of fidelity in signal processing. 

Moving from the retina to central areas of the visual system, extensive research efforts are currently devoted to understanding the molecular and cellular mechanisms involved in the control of physiological and pathological developmental trajectories at the visual cortex level. A class of molecules particularly relevant in this field are matrix metalloproteinases (MMPs), enzymes capable of interacting with a network of proteins called the extracellular matrix and contributing to both visual cortex development and plasticity in young animals and to visual plasticity in adulthood. Murase et al. [11] combined the genetic deletion of MMP9 with chronic monocular deprivation, and used calcium imaging of supra-granular neurons in the primary visual cortex to monitor the effects of this combined treatment. While, in wild-type mice, chronic deprivation initiated at eye opening significantly decreased the strength of deprived-eye visual responses and affected orientation and direction selectivity, these changes did not occur in MMP9 ^−/−^ mice raised with conventional binocular vision. However, MMP9^−/−^ mice turned out to be insensitive to visual cortex plasticity in response to chronic deprivation, while they appeared totally normal when considering other forms of experience-dependent plasticity, such as the well-established mechanism of stimulus-selective response potentiation [12]. These results will help to expand the knowledge concerning the molecular specificity of distinct forms of activity-dependent plasticity in the mouse visual system. 

Among the possible experimental approaches capable of promoting brain plasticity, particularly promising are those based on non-invasive procedures that can enhance the potential for plasticity retained by neural circuitries without being associated with dangerous side effects. Poh et al. [13] used repetitive transcranial magnetic stimulation (rTMS), a non-invasive brain stimulation technique, asking whether the beneficial effects of this treatment may be dependent on the subject’s brain state at the time of stimulation. To monitor the impact of rTMS, the authors employed mice with homozygous null mutations of the ephrin-A2 and -A5 genes, which are known to exhibit aberrant retinotectal, geniculocortical and corticotectal projections, and which have been previously shown to be susceptible to the effects of rTMS [14]. By administering chronic rTMS in adult ephrin-A2A5^−/−^ mice either in a dark environment or concurrently with voluntary locomotion, the authors reported that while rTMS was able to refine the geniculocortical map in either treatment condition, corticotectal projections were improved only when the stimulation paradigm was combined with physical exercise, without any beneficial effect in the group exposed to dark rearing. 

One of the most astonishing features of brain plasticity is its capability to drive the recovery of lost functions following pathologies or brain injuries. In the visual system, reorganizations after cortical damage leading to recovery of the subsequent scotomas in the visual field are well documented at early ages, but much less in adulthood. Bath et al. [15] described an impressive case of highly preserved residual visual-perceptual capacities in a 12-year-old girl with a congenital and extensive brain lesion in the left hemisphere, along with a microphthalmos in the left eye and missing contralateral lateral geniculate nucleus of the thalamus. The authors noted that despite the lack of inputs to the affected side of the striate cortex, the patient had a very diffuse scotoma, suggesting extensive plasticity and the reorganization of visual pathways. Strikingly, an assessment of contrast sensitivity and retinotopic organization of the patient revealed nearly normal visual functions, with respect to healthy subjects, suggesting the occurrence of a dramatic redirecting of the visual information necessary to achieve a normal cortical retinotopic representation in the primary visual cortex. To achieve a better understating of the compensatory mechanisms underlying the perceptual abilities of the patient, the authors performed a structural assessment with white matter diffusion tractography, revealing a marked reorganization of the thalamus and tectum in the intact hemisphere.

My hope is that this Special Issue will contribute to emphasizing the relevance of mechanistic studies aimed at uncovering major cellular and molecular factors underlying visual system plasticity under both physiological and pathological conditions. This area of investigation will likely remain essential as one of the most successful in the field of brain repair.
